# Next-Generation Sequencing in Critically Ill COVID-19 Patients with Suspected Bloodstream Infections: A Retrospective Cohort Study

**DOI:** 10.3390/jcm12041466

**Published:** 2023-02-12

**Authors:** Christoph J. Leitl, Sandra E. Stoll, Wolfgang A. Wetsch, Tobias Kammerer, Alexander Mathes, Bernd W. Böttiger, Harald Seifert, Fabian Dusse

**Affiliations:** 1 Department of Anesthesiology and Intensive Care Medicine, Faculty of Medicine, University Hospital of Cologne, University of Cologne, Kerpener Straße 62, 50937 Cologne, Germany; 2 Institute for Medical Microbiology, Immunology and Hygiene, Faculty of Medicine, University Hospital of Cologne, University of Cologne, Goldenfelsstraße 19-21, 50935 Cologne, Germany

**Keywords:** blood culture, sepsis, antimicrobial therapy, bacteremia, DISQVER^®^

## Abstract

Background: Rapid pathogen identification and appropriate antimicrobial therapy are crucial in critically ill COVID-19 patients with bloodstream infections (BSIs). This study aimed to evaluate the diagnostic performance and potential therapeutic benefit of additional next-generation sequencing (NGS) of microbial DNA from plasma in these patients. Methods: This monocentric descriptive retrospective study reviewed clinical data and pathogen diagnostics in COVID-19 ICU patients. NGS (DISQVER^®^) and blood culture (BC) samples were obtained on suspicion of BSIs. Data were reviewed regarding the adjustment of antimicrobial therapy and diagnostic procedures seven days after sampling and analyzed using the Chi²-test. Results: Twenty-five cases with simultaneous NGS and BC sampling were assessed. The NGS positivity rate was 52% (13/25) with the detection of 23 pathogens (14 bacteria, 1 fungus, 8 viruses), and the BC positivity rate was 28% (7/25, 8 bacteria; *p* = 0.083). The NGS-positive patients were older (75 vs. 59.5 years; *p* = 0.03) with a higher prevalence of cardiovascular disease (77% vs. 33%; *p* = 0.03). These NGS results led to diagnostic procedures in four cases and to the commencement of four antimicrobial therapies in three cases. Empirical treatment was considered appropriate and continued in three cases. Conclusions: In COVID-19 patients with suspected BSIs, NGS may provide a higher positivity rate than BC and enable new therapeutic approaches.

## 1. Introduction

Since 2020, the coronavirus disease-2019 (COVID-19) has posed a serious burden on the global healthcare system and especially on intensive care units (ICUs). The progressive availability of vaccines, the emergence of less virulent strains, the growth of clinical experience, and the development of new treatments effectively decreased the number of ICU admissions and overall mortality rates over the course of the pandemic [1,2,3,4]. However, in critically ill patients requiring invasive mechanical ventilation, mortality rates remained high throughout the later waves of the pandemic [5,6]. In particular, secondary infections, such as bloodstream infections (BSIs) are strongly associated with poorer outcomes [7,8,9].

Delayed or inadequate antimicrobial treatment is associated with increased morbidity and mortality rates in sepsis [10,11,12]. Consequently, the rapid initiation of empiric antimicrobial therapy and the identification of the causative pathogen is crucial.

However, conventional, culture-based methods—which form the current gold-standard for pathogen identification—suffer from limitations, such as delayed results and low test sensitivity, especially with previous exposure to antibiotics [13,14,15]. Polymerase chain reaction (PCR)-based techniques have been developed as rapid alternatives to culture-based methods, but these approaches often rely on targeted pathogen detection with limited coverages [16].

Recently, next-generation sequencing (NGS)-based methods have emerged as powerful diagnostic platforms for the detection of pathogens in critically ill patients [13,17,18]. The concept of unbiased sequence analysis of circulating cell-free deoxyribonucleic acid (cfDNA) from plasma allows for the identification of bacterial, fungal, and viral microorganisms in one single test, including non-culturable pathogens (e.g., *Tropheryma whipplei* or *Coxiella burnetii*) and irrespective of antimicrobial treatment. In particular, the DISQVER^®^ pathogen test (Noscendo GmbH, Duisburg, Germany) provides comprehensive data analysis and allows differentiation between relevant pathogens and potential microbial contaminants, such as coagulase-negative staphylococci (CNS), by calculating a sepsis-indicating quantifier score [19]. Previous studies have shown a higher sensitivity of NGS-based methods compared to blood cultures (BCs) in patients with suspected sepsis or BSIs, which is potentially beneficial for the optimization of antimicrobial treatments [13,17,18]. However, to date, only a few studies have addressed the clinical impact of complementary NGS diagnostics in patients with either suspected sepsis or BSIs [13,20].

Given the high rate of secondary infections and associated increased mortality in patients with severe COVID-19, we hypothesized that this group in particular would benefit from improved pathogen diagnostics. The aim of this descriptive study was to evaluate the diagnostic performance of NGS-based methods and their potential impact on antimicrobial therapy in a cohort of critically ill COVID-19 patients. To the best of our knowledge, this is the first study investigating the implementation of this new approach in the diagnosis of BSIs in patients with severe COVID-19.

## 2. Materials and Methods

### 2.1. Setting and Patients

This retrospective, observational study was conducted between November 2020 and March 2021 at a German 14-bed COVID-19 ICU (Department of Anesthesiology and Intensive Care Medicine, University Hospital of Cologne, Germany). Included in this study were adult patients (age ≥ 18 years) with confirmed SARS-CoV-2 infections requiring ICU treatment. A confirmed SARS-CoV-2 infection was defined as a positive reverse transcriptase PCR result obtained from nasopharyngeal swabs and/or lower respiratory tract aspirates. Samples for BC and NGS were obtained when a BSI was suspected by the attending physician based on the clinical signs and symptoms of sepsis. The study was approved by the Ethics Committee of the Medical Faculty of the University of Cologne (Reference No. 21-1444).

### 2.2. Blood Cultures

Blood samples were obtained either via sterile venipuncture or from a central venous catheter (CVC) after thorough disinfection, according to the institutional standard. At least two pairs of BCs (aerobic and anaerobic, volume 8–10 mL each) were obtained and inoculated (BACTEC, Becton Dickinson, Heidelberg, Germany). The BC bottles were sent to the institutional laboratory and analyzed as previously described [21]. Samples were incubated for up to seven days and the institutional average time to positivity for this method was 13 h.

### 2.3. Next-Generation Sequencing

Blood samples were drawn under the same conditions as mentioned above, collected into stabilizing blood tubes (Cell-Free DNA BCT CE, Streck, La Vista, NE, USA), and shipped at ambient temperature by a medical logistics service provider to a specialized laboratory (Noscendo GmbH, Reutlingen, Germany). Blood samples were separated to plasma by centrifugation at 1600× *g* for 10 min at 4 °C and the plasma supernatant was transferred to a fresh reaction tube. Then, a second centrifugation step at 16,000× *g* for 10 min at 4 °C was performed, supernatants were again transferred, and plasma aliquots were further stored. Nucleic acid isolation, quality controls, and library preparation were carried out as previously described [22]. Adequate positive and negative controls accompanied all laboratory and sequencing procedures. All data generated were analyzed using Noscendo’s DISQVER^®^ platform, which comprises a curated microbial genome reference database of over 16,000 microbial species, including bacteria, DNA viruses, fungi, and parasites, while potential contaminations and commensals were discriminated from infective agents based on statistical calculations [23]. The analysis time for this method is specified as less than 24 h after the sample is received by the laboratory. Reports were accessible to the treating clinician via an online portal after email notification.

### 2.4. Virology

Additional diagnostic tests for viruses from blood samples were only conducted if viral DNA was detected by NGS and considered as potentially clinically relevant, or if a viral infection was clinically suspected by the attending physician. The routine virus detection panel for blood samples was performed by real-time PCR, as per institutional protocol, and included herpes simplex virus type 1 and 2 (HSV-1/2), Epstein–Barr virus (EBV), and cytomegalovirus (CMV).

### 2.5. Data Collection

Data were collected retrospectively through a standardized case report form from electronic and paper medical records. Data included patient demographics, length of ICU and hospital stay, major comorbidities, and discharge data. Clinical data obtained on admission and on the day of sample collection included relevant laboratory data, such as C-reactive protein (CRP), procalcitonin (PCT), and leucocytes, as well as the Sequential Organ Failure Assessment (SOFA) score, therapeutic measures, such as mechanical ventilation, renal replacement therapy, and vasopressor support, antimicrobial treatment, and infectious source control measures. Results from other routine microbiological tests (tracheal secretions, drainage samples, urine, orsamples from surgical sites) were performed within three days, either, before or after blood sample collection for NGS diagnostics were included in the evaluation. Changes in antimicrobial therapy and infectious source control procedures within seven days of NGS sampling were reviewed. Additionally, in patients with viruses detected by NGS, medical records were screened for virological tests and antiviral therapies.

### 2.6. Data Review

A panel composed of at least two intensivists and one microbiology specialist examined medical files, including clinical parameters, previous course of the disease, consultations with infectious disease specialists, antimicrobial treatments, and results from pathogen diagnostics. Results of the NGS analysis were assessed for clinical relevance and categorized as to whether the results (1) provided any additional or unique findings, (2) led to further diagnostic measures, or (3) affected antimicrobial therapy. The therapeutic impact was further distinguished between (I) initiation of additional antimicrobial treatment, (II) confirmation of therapy already initiated, or (III) discontinuation of ongoing antimicrobial treatment. Identification of typical BC contaminants, such as CNS, was assessed for clinical relevance. Contamination was assumed if the suspected isolates were considered as such, either according to clinical documentation or were present in only one BC and no further action was taken.

### 2.7. Statistics

Statistical analysis was performed using IBM SPSS statistics software version 28.0 (IBM Corp., Armonk, NY, USA). Continuous data are presented as the median and interquartile range, categorial data are presented as counts and percentages. Quantitative variables were compared using the Mann-Whitney test, qualitative variables were compared using the Chi-square test or Fisher’s exact test, as appropriate. A *p*-value < 0.05 was considered statistically significant.

## 3. Results

### 3.1. Study Population

A total of 25 cases with simultaneous BC and NGS sampling were identified for analysis. Demographic data and clinical variables are presented in Table 1. The median (IQR) age was 70 years (56.5–76.5) and patients were predominantly male (16/25, 64%). Patients with positive NGS results were older (75 vs. 59.5 years; *p* = 0.03) and a history of cardiovascular disease was more common in these patients (77% vs. 33%; *p* = 0.03). Other demographical or clinical variables were similar in cases with positive or negative NGS results. The median (IQR) length of ICU stay was 20 days (11–33.5), and in-hospital mortality was 64% (16/25). At the time of sampling, the median (IQR) SOFA score was 8 (6.5–10.5) with 52% of patients (13/25) requiring invasive mechanical ventilation and 88% of patients (22/25) depending on vasopressor support. Antimicrobial therapy was administered in 56% of cases (13/25) and did not significantly affect NGS or BC positivity rates (NGS *p* = 0.32, BC *p* = 0.67). All samples were collected ≥ 48 h after hospital admission.

### 3.2. NGS and BC Results

Results from 25 NGS tests and 61 sets of BC (minimum of two sets per case) from 25 COVID-19 ICU patients with suspected BSIs were assessed. At least one isolate was detected by NGS or BC in 64% of cases (16/25) and the combination of NGS/BC found 31 microorganisms, including 22 bacteria, 1 fungus, and 8 viruses. An overview of all detected isolates is presented in Table 2.

Accounting for all pathogens, NGS showed a statistically non-significant higher positivity rate than BC (NGS: 52%, 13/25 vs. BC: 28%, 7/25; *p* = 0.083). NGS identified 23 isolates in total (14 bacteria, 1 fungus, and 8 viruses), whereas BC only detected eight bacterial species (*p* = 0.20).

Contamination of positive samples was less frequent in NGS (15%, 2/13) than in BC (57%, 4/7; *p* = 0.12). Contaminants found in NGS were *Xanthomonas campestris* (*n* = 1) and *Corynebacterium imitans* (*n* = 1), whereas contamination in BC was only caused by CNS (*n* = 5). These isolates were excluded from the analysis.

The sensitivity for potentially relevant pathogens for NGS and BC combined was 48% (12/25). A comparison of NGS and BC regarding potentially clinically relevant results revealed that NGS provided significantly more positive results than BC (*p* = 0.01). NGS returned positive results in 48% of cases (12/25) and identified 12 bacteria, 1 fungus, and 8 viruses. Three bacteria considered relevant were detected by BC in 12% of cases (3/25).

### 3.3. Direct Comparison of NGS and BC

Excluding viruses from NGS results for a more direct comparison, positive results were detected in 36% of cases (9/25) by NGS and in 12% of cases (3/12) by BC (*p* = 0.05). Both methods returned positive results in three cases and agreed on two bacterial species (*Escherichia coli* and *Enterococcus faecium*). In the remaining specimen, NGS and BC provided inconsistent findings (NGS: *Staphylococcus aureus*, *E. faecium*, *Serratia marcescens*; BC: *Staphylococcus epidermidis*). In nine cases with positive NGS results (seven bacteria, one fungus, and seven viruses), BC analysis remained negative. In 13 cases, no clinically relevant pathogen was found by either method.

### 3.4. Diagnostic Benefit of NGS

Compared to BC alone, additional NGS diagnostics provided further information in 44% of cases (11/25). NGS identified ten bacteria and one fungus that remained undetected in simultaneously collected BC samples.

Even when including results from other routine microbiological tests, such as surgical swabs, tracheal secretions, and urine, NGS identified four bacteria and one fungus, which were not found in conventional diagnostics (*E. faecium*, *n* = 2; *Bacteroides fragilis*, *n* = 1; *Helicobacter pylori*, *n* = 1; *Candida parapsilosis*, *n* = 1). NGS confirmed a systemic infection in four cases by detecting six bacteria in the bloodstream, which were otherwise only identified in samples taken directly from the septic focus (abdomen, *n* = 1, *E. faecium*, *Enterococcus. raffinosus*; lung *n* = 3, *E. coli*, *S. marcescens*, *S. aureus*, *Klebsiella pneumoniae*).

Since routine screening for viraemia was not routinely performed, NGS revealed eight viral isolates in seven specimens (EBV: *n* = 4; HSV-1: *n* = 2; CMV: *n* = 2), which would otherwise have remained undetected.

### 3.5. Additional Viral Diagnostic

In four out of seven cases with positive viral NGS, additional PCR confirmed four out of five viruses. EBV and HSV-1 were each confirmed in two out of two cases by PCR. Additionally, HSV-1 was detected by PCR in two further samples, which were missed by NGS. In one patient, positive HSV-1 NGS results led to the suspicion of herpes simplex encephalitis, which was consequently excluded by PCR from cerebrospinal fluid after lumbar puncture.

### 3.6. Antimicrobial Therapy

**Figure 1:**
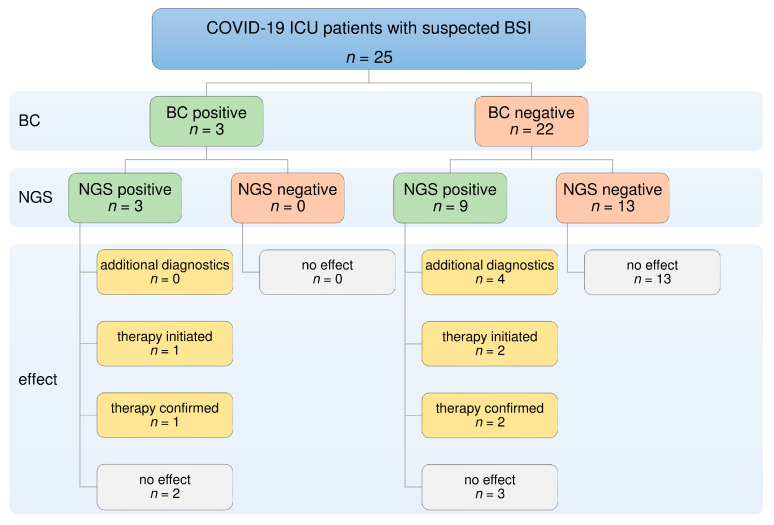
Diagnostic and therapeutic contribution of additional NGS considering BC positivity. BC failed to detect relevant pathogens in 22 of 25 cases. In six of these cases, additional NGS led to further diagnostic tests or contributed to the optimization of antimicrobial therapy. BC: blood culture; BSI: bloodstream infection; NGS: next-generation sequencing.

Results from additional NGS analysis affected therapy in 20% of all cases (5/25) (Table 3, Figure 1). Identification of clinically relevant pathogens by NGS led to the initiation of four antimicrobial therapies in three cases:

(1) Aciclovir was administered in two patients following positive NGS results for HSV-1, with confirmation by PCR.
(2) Vancomycin treatment was started in two patients after the detection of *E. faecium* by NGS.

Besides clinical factors, NGS further contributed to the continuation of empirical antimicrobial therapy in three cases. Considering the lack of antimicrobial susceptibility testing, empirical antimicrobial treatment was assumed to be appropriate after NGS provided the only identification of causative pathogens in blood samples. NGS did not substantially contribute to the termination of therapy in any of these cases.

## 4. Discussion

This retrospective study investigated the diagnostic performance and potential impact on antimicrobial therapy of additional NGS pathogen diagnostics in COVID-19 ICU patients. NGS was able to detect three times as many bacterial or fungal pathogens in blood samples compared to BC alone (*p* = 0.05). Moreover, NGS detected a viral reactivation in 28% of patients, which would have remained undiscovered by standard diagnostic alone. Based on these results, NGS directly led to the initiation of targeted antimicrobial therapy in 12% of all cases and contributed to the continuation of appropriate therapy in 12% of cases, considering the overall clinical context. NGS led to additional diagnostic procedures, which potentially altered therapy.

In this study, bacterial BSIs were diagnosed by BC analysis in only 12% of cases despite corresponding clinical symptoms. This may be due to the limitations faced by classical culture-based methods: (1) low sensitivity, especially for slow-growing and fastidious organisms, (2) interference due to prior antibiotic exposure, (3) contamination often led to false positive results, and (4) long turnaround time, as standard culture methods typically require 12–36 h for positive signaling and up to 72 h for accurate pathogen identification, including antimicrobial susceptibility testing [13,14,15].

NGS analysis detected ten additional bacteria as well as one fungus compared to BC in this study cohort. Furthermore, six bacteria were detected in the bloodstream, which otherwise would have been regarded as localized infections (i.e., isolates were only detected in routine non-blood samples). Previous studies reported a 1.5–5.2-fold increase in sensitivity by NGS in patients with suspected sepsis compared to BC [13,17,18]. Our results are in line with these previous studies and demonstrate a higher positivity rate of NGS in the detection of bacteria or fungi compared to BC analysis, in COVID-19 patients (36% vs. 12%, *p* = 0.05).

### 4.1. Confirmation of Positive BC Results by NGS

In only 3 of 25 cases were relevant pathogens detected by both BC and NGS. BC confirmed NGS results in two cases, yet was unable to detect one clinically relevant bacterium in one patient: *S. epidermidis* was isolated in two sets of BCs drawn from a CVC in one patient, whereas NGS detected three different bacteria. These BC results were considered catheter-related BSI according to clinical documentation and, consequently, the CVC was removed, and ongoing antimicrobial treatment continued.

A putative lack of pathogens in NGS analysis might be explained by a technical analysis algorithm. As described in a previous study, isolates might have been identified by NGS but not reported due to a low read count and stringent threshold settings during analysis [13]. Thus, low concentrations of pathogens prevent further analysis and might lead to disagreement between the two methods [24]. Further research will be necessary to address this question and possibly improve analysis algorithms.

### 4.2. Defining Antimicrobial Therapy Using NGS

This study examined the potential impact of NGS results on the choice of antimicrobial treatment. In three cases, four antimicrobial therapies were initiated following positive NGS results. In addition, NGS found pathogens in twelve cases, in which BC remained negative. In three of those cases, empirical therapy was deemed appropriate, and no adjustment seemed necessary. In some cases, the identification of additional pathogens in the bloodstream might have even wider implications, as demonstrated in one case. While conventional methods only found *S. aureus* in respiratory samples and BC remained negative, NGS detected *S. aureus* DNA in the bloodstream. This could have warranted a prolonged duration of antimicrobial therapy and further diagnostic procedures, such as echocardiography to assess for endocarditis. Although this study was not designed to demonstrate a significant benefit of NGS regarding therapy improvement, it can be hypothesized that additional NGS diagnostics may lead to the optimization of antimicrobial therapy in certain critically ill COVID-19 patients. However, given the high cost of NGS compared to standard diagnostics and the still unclear overall limited therapeutic benefit, the indication for the use of NGS should be carefully considered.

### 4.3. Contamination

An unusually high rate of contamination by CNS in BC diagnostics was observed in this study: CNS were detected in 16% of all specimens and in 57% of positive specimens, which is significantly higher than the usual false-positivity rate reported in our annual pathogen and resistance statistics. In two extensive reviews, performed before SARS-CoV-2 emerged, the overall contamination rates of BC were notably lower and ranged from only 0.6 to 12.5% [25,26]. During the pandemic, a general increase in contamination rate in specimens from COVID-19 individuals was observed, presumably caused by a high workload, newly trained staff, wearing full personal protective equipment, and time pressures [27,28,29,30]. Since this study was conducted during the peak of the second and third waves of the pandemic, these aspects may also have been major contributors to this study. In contrast, no CNS were identified in any specimen by NGS. This circumstance may reflect methodological differences. NGS analysis only targets cell-free DNA released by degradation processes or immune system interaction. In cases of contamination, bacterial cells remain mostly intact, avoiding DNA release, which consecutively leads to negative NGS results. This clearly differs from the BC methodology, in which vital bacteria are cultivated followed by positive signaling.

### 4.4. Value of NGS in the Diagnosis of Fungal Infections

Critically ill COVID-19 patients are at increased risk of developing secondary fungal infections such as COVID-19-associated pulmonary aspergillosis and candidemia [31,32,33,34,35,36]. In this study, one isolate of *C. parapsilosis* was found only by NGS. However, the result was not considered clinically relevant. According to current guidelines, the diagnosis of candidemia could have warranted additional interventions, such as CVC removal and ophthalmological examination [37]. However, recent studies reported inconsistent results regarding the benefit of NGS in the detection of systemic fungal infections and larger prospective studies will be needed to assess this question [13,17,18,24].

### 4.5. Value of NGS in the Diagnosis of Viral Infections

Reactivation of latent viruses is common in patients with sepsis and may be even more frequent in patients with severe COVID-19 [38,39,40,41,42,43]. However, tests for viral infections in clinical routines are lacking. In our study, the implementation of additional NGS analysis led to the identification of 8 viruses in 25 patients, which would have been missed by standard diagnostics. However, in the absence of a clinically apparent viral disease in most cases, the clinical relevance and therapeutic implications of these results remain unclear.

In two patients, treatment with aciclovir was initiated following the identification of HSV-1 by NGS. However, data regarding the prognostic implications of HSV reactivation in patients with COVID-19 is inconclusive, while the only randomized controlled trial on aciclovir treatment in non-COVID-19 patients found no benefit on morbidity or mortality [31,39,40,44,45]. Whether viral reactivation of HSV or CMV in critically ill patients reflects true viral disease (and, therefore, represents possible treatment strategies), or is merely indicative of an immunocompromised state remains controversial and requires further investigation.

### 4.6. Methodological Characteristics and Limitations of NGS

The relevance of the microorganisms detected by NGS often remains unclear. Clinical experience and treatment recommendations are limited or lacking. Similar to other molecular genetic detection methods, it is uncertain whether the detection of cfDNA corresponds to clinically relevant infection. The genomic material obtained could originate from non-viable or commensal microorganisms and, therefore, might lead to false positive results and mimic an active infection. Furthermore, molecular techniques, currently do not allow for antimicrobial susceptibility testing [16]. In our institution, NGS currently offers no advantage over culture-based diagnostics in terms of turn-around time for logistic reasons. Improved workflows that might provide faster results in the future are currently under investigation [23]. Results of prospective studies showing a positive effect of NGS on clinical outcomes and, thus, justifying the additional costs are still pending [22].

Identifying microorganisms of uncertain clinical relevance and lack of antimicrobial susceptibility testing can result in antimicrobial overtreatment and prevent de-escalation and rational use of antibiotics. These technical limitations demonstrate that NGS-based analysis cannot be used as a substitute for cultural methods, but rather may be considered as a complementary test in patients with severe COVID-19.

### 4.7. Limitations

This study suffers from numerous limitations, which are mainly attributable to the retrospective study design and the limited cohort. As such, the study design was not suitable to investigate the impact of additional NGS-based diagnostics on morbidity and mortality. A very specific subgroup of patients with severe COVID-19 treated in the ICU was examined, consequently, our results cannot be generalized to other patient groups. The lack of routine screening for common viral infections impedes any direct comparison of the two methods, while sample collection from different sites could lead to divergent results between the methods. Significant differences in demographic variables, such as age or pre-existing diseases, could constitute confounding factors and the small number of individual pathogens precluded an analysis of the association between read count, outcome, and clinical relevance. Despite a comprehensive review of clinical data and documentation, important information may have been unavailable, potentially biasing the evaluation of treatment decisions.

## 5. Conclusions

The results of our study suggest that NGS-based diagnostics might offer a higher positivity rate than conventional culture-based methods and, therefore, may enable new therapeutic approaches in critically ill COVID-19 patients. However, further experience regarding the interpretation of the results is required and treatment decisions should be carefully considered to avoid overtreatment. Larger, prospective studies will be necessary to determine whether the identification of additional pathogens by NGS can improve the outcome of critically ill ICU patients with severe COVID-19.

## Figures and Tables

**Table 1:** Demographic data and clinical variables at the time of sampling. Demographic and clinical data showed no relevant differences regarding NGS positivity. Data are presented as the median and interquartile range or as counts and percentages, as appropriate. CRP: C-reactive protein; ICU: intensive care unit; IL-6: interleukin 6; NGS: next-generation sequencing; PCT: Procalcitonin; SOFA: sequential organ failure assessment.

Variable	Total (*n* = 25)	NGS		*p*
		Positive (*n* = 13)	Negative (*n* = 12)	
Age (years)	70.0 (56.5–76.5)	75.0 (70.0–78.5)	59.5 (49.5–71.5)	0.03
Male sex	16 (64)	8 (62)	8 (67)	1
Admission source				
Emergency room	11 (44)	6 (46)	5 (42)	0.82
General hospital ward	7 (28)	4 (31)	4 (33)	0.67
Intermediate care unit	1 (4)	-	1 (8)	0.48
Intensive care unit	6 (24)	3 (23)	2 (17)	0.65
ICU stay (days)	20.0 (11.0–33.5)	22.0 (11.0–44.5)	20.0 (11.3–29.0)	0.61
Mechanical ventilation (days)	13.0 (8.5–27.0)	18.0 (9.0–39.0)	12.0 (4.75–21.3)	0.34
In-hospital death	16 (64)	9 (69)	7 (58)	0.69
Comorbid conditions				
Arterial hypertension	18 (72)	10 (77)	8 (67)	0.67
Cardiovascular disease	14 (56)	10 (77)	4 (33)	0.03
Pulmonary disease	4 (16)	3 (23)	1 (8)	0.59
Renal disease	3 (12)	2 (15)	1 (8)	1
Diabetes mellitus	5 (20)	3 (23)	2 (17)	1
Status at sampling				
SOFA-Score	8.0 (6.5–10.5)	9.00 (6.0–11.5)	7.50 (6.3–8.0)	0.43
Ventilation				
Oxygen support	8 (32)	2 (15)	6 (50)	0.10
Non-invasive ventilation	4 (16)	2 (15)	2 (17)	1
Invasive mechanical ventilation	13 (52)	9 (69)	4 (33)	0.07
Oxygenation (paO_2_/FiO_2_, mmHg)	144.0 (94.5–183)	144.0 (103–195)	144.0 (87.8–184)	0.74
Vasopressor therapy	22 (88)	12 (92)	10 (83)	0.59
Renal replacement therapy	8 (32)	6 (46)	2 (17)	0.20
Antimicrobial therapy	13 (52)	8 (62)	5 (42)	0.32
Laboratory values				
Leucocytes (10^9^/L)	12.4 (8.36–16.5)	12.2 (8.71–18.1)	12.48 (8.27–15.4)	1
Neutrophils (10^9^/L)	8.13 (7.20–14.6)	8.13 (7.57–14.8)	9.430 (5.74–13.8)	0.74
Lymphocytes (10^9^/L)	0.63 (0.46–1.33)	0.72 (0.48–1.60)	0.62 (0.42–1.09)	0.36
CRP (mg/L)	154 (82.2–219)	130 (66.9–203)	164 (120–252)	0.25
PCT (µg/L)	1.00 (0.40–3.60)	2.20 (0.45–5.45)	0.50 (0.23–1.38)	0.07
IL-6 (ng/L)	82.0 (35.0–686)	109 (35.0–686)	76.0 (34.0–869)	0.87

**Table 2:** Microorganisms detected by NGS, BC, and PCR. NGS detected 23 isolates (14 bacteria, 1 fungus, and 8 viruses) and provided positive results in 52% (13/25) of cases, whereas BC identified eight bacteria in 28% (7/25) of cases. The most frequently detected bacteria were Enterococcus species in NGS and coagulase-negative staphylococci in BC. Following the identification of viruses by NGS, PCR confirmed four out of five viruses and detected two further isolates of HSV-1. ^†^ Coagulase-negative staphylococci. BC: blood culture; HSV-1: herpes simplex virus type 1; NGS: next-generation sequencing; PCR: polymerase chain reaction.

Microorganism	NGS (*n* = 25)	BC (*n* = 25)
Bacteria	14	8
*Enterococcus faecium*	4	1
*Escherichia coli*	2	1
*Enterococcus raffinosus*	1	-
*Serratia marcescens*	1	-
*Klebsiella pneumoniae*	1	-
*Staphylococcus aureus*	1	-
*Helicobacter pylori*	1	-
*Bacteroides fragilis*	1	-
*Staphylococcus epidermidis* ^†^	-	1
considered as contaminant		
*Staphylococcus epidermidis* ^†^	-	3
*Corynebacterium imitans*	1	-
*Xanthomonas campestris*	1	-
*Staphylococcus hominis* ^†^	-	1
*Staphylococcus capitis* ^†^	-	1
Fungi	1	-
*Candida parapsilosis*	1	-
	NGS (*n* = 25)	PCR (*n* = 4)
Viruses	8	6
Epstein–Barr virus	4	2
Herpes simplex virus type 1	2	4
Cytomegalovirus	2	-

**Table 3:** Contribution of NGS to the optimization of antimicrobial therapy. NGS results led to the initiation of four targeted therapies in three cases. Aciclovir was administered in two cases after HSV-1 was detected by NGS and confirmed by PCR. Vancomycin was started in two cases following the detection of E. faecium by NGS. In three cases, following pathogen detection by NGS, ongoing empiric therapy was considered appropriate and was continued accordingly. BAL: bronchoalveolar lavage; BC: blood culture; EBV: Epstein–Barr virus; HSV-1: herpes simplex virus type 1; NGS: next-generation sequencing; PCR: polymerase chain reaction.

ID	Age/Sex	(Suspected) Source of Infection	Diagnostic Method			Antimicrobial Therapy	
			NGS	BC	Other	Empiric	Contribution of NGS
N3	79/f	Pulmonary	*X. campestris*, HSV-1	Negative	Serum (PCR): HSV-1	Meropenem	Initiation of aciclovir treatment
N8	70/m	Pulmonary	*B. fragilis*, EBV	Negative	Serum (PCR): EBV	Piperacillin/tazobactam	Confirmation of empiric therapy
N12	78/m	Pulmonary	*E. faecium*, HSV-1, EBV	Negative	Serum (PCR): HSV-1	Meropenem	Initiation of vancomycin and aciclovir treatment
N19	31/m	Unknown	*S. aureus*, *S. marcescens*, *E. faecium*	*S. epidermidis*	BAL: *S. aureus*	Meropenem	Initiation of vancomycin treatment, confirmation of empiric therapy
N25	80/f	Pulmonary or wound	*K. pneumoniae*	Negative	BAL: *K. pneumoniae*	Meropenem	Confirmation of empiric therapy

## Data Availability

The datasets used and analyzed during the current study are available from the corresponding author upon reasonable request.
